# *Neurobiology of Language*: Editorial

**DOI:** 10.1162/nol_e_00009

**Published:** 2020-03-01

**Authors:** Steven L. Small, Kate E. Watkins

**Affiliations:** School of Behavioral and Brain Sciences, University of Texas at Dallas, USA; Department of Experimental Psychology, University of Oxford, UK

## OVERVIEW

Welcome to the first issue of the first volume of the *Neurobiology of Language*. As co-editors-in-chief, we thought this first editorial should explain our reasons for launching a new open access journal and describe our ambition for its future.

### Background of the Field

The non-invasive measurement of brain signals at high resolution has fundamentally changed research into the biological basis of behavior. This is particularly true for the study of language, in which traditional neuroscience approaches involving animal models are not viable. Spatially and temporally precise methods of interrogating the brain permit serious investigation of the neurobiology of language and the linking together of conceptual premises from psychology, linguistics, and neuroscience.

Although investigations of the neurobiology of language focus primarily on brain mechanisms, emphasizing questions in systems and cellular neuroscience, genetics, anatomy, physiology, and evolution, such work also requires substantial knowledge of psychology and linguistics. Given the unique nature of human language, the neurobiology of language is primarily a field involving human subjects and has benefited from the novel and highly robust methods of brain measurement. Nevertheless, the field also adopts the central tenets of modern biology, including the evolutionary imperative that the human brain evolved from a common ancestor, and thus the study of other species plays an important role in the investigations of the neurobiology of language. The journal aims to be the most important venue for scholars interested in this area, with a broad scope of disciplinary topics, representing not only neurobiological data but also data from all related disciplines that would have direct relevance to scientists studying the biology of language.

Our field thus involves highly interdisciplinary inquiry to integrate insights from the full range of basic scientific fields that are concerned with human language, including (but not limited to) neuroscience, linguistics, psychology, genetics, motor control, and computational modeling, and clinical fields such as neurology and speech pathology. The availability of advanced technologies and high-performance computing has not only opened up exciting new avenues of scientific study and re-energized existing research fields, but also expanded the set of required skills and knowledge for language researchers engaged in this type of research. It is thus of utmost importance to foster dialogue and interaction among the relevant disciplines to continue to shape this substantially re-created area of research and promote scientific investigation of the highest quality.

### An Emerging Community

The first conference on the neurobiology of language was held in Chicago in 2009 as a satellite symposium of the annual meeting of the Society for Neuroscience. This first meeting clearly underlined the need for such a new forum by attracting 350 participants from myriad scientific backgrounds and more than 20 different countries. The success of the first meeting was not anomalous: Participation has steadily increased, with more than 600 researchers present at each of the last three meetings (London, Baltimore, Helsinki). The early success led to the founding in 2011 of the Society for the Neurobiology of Language (SNL) to empower the community to actively shape the emerging discipline that aims above all to understand how the brain does language.

### A New Journal

We first discussed the idea of a new journal early in 2017. Both of us had several years’ experience editing manuscripts for the journal *Brain and Language*: Steven Small was editor in chief there for almost 15 years; Kate Watkins was an action editor for several years and later also one of the Language section editors for *Neuropsychologia*. Both are active researchers in the field with experience of publishing and reviewing in many other outlets. Part of our motivation for wanting a new outlet was driven by dissatisfaction with large commercial publishers, whose business model consists of charging subscription fees to readers of articles about research that is largely funded by governments and charitable organizations and peer-reviewed by unpaid volunteer scientists. In recent years, these costs have become prohibitive for many individuals and institutions, despite increasing public interest in scientific results. The lack of access by taxpayers to research that they have funded has been particularly striking. Furthermore, we felt that editors, authors, and peer reviewers, who contributed considerable time and effort to the editorial process were not being supported well in terms of the infrastructure for publishing.

## SOCIETY FOR THE NEUROBIOLOGY OF LANGUAGE

Both editors-in-chief are supporters and active members of SNL. At the time of writing, the society has completed eleven annual conferences, with an annual attendance well in excess of 500 individuals, with over 1,500 individuals attending at least one meeting. The success of the society is an indicator of the health of the field, and it was precisely this community that we felt would be well served by a new journal such as this. The annual meeting of the society was already supported by two journals—*Brain and Language* and *Language, Cognition and Neuroscience*—but both are subscription-based commercial publications, and neither is fully open access. Prior to the 2017 meeting in Baltimore, all SNL members were asked to complete an online survey about launching a new journal. The results were very positive, including strong support for open access, not-for-profit publication, unbiassed and modern publishing policies, and affiliation with the society. We were enthused by the clear support and appetite for the proposal from society members. We also received encouragement from the society itself that they were keen to pursue this idea with us and have a society-owned journal.

## MOTIVATION FOR THE PROJECT: BROAD STROKES

There are many reasons that we believed that the time was right to develop a new journal, and this is certainly not because of the paucity of journals—even reputable ones, which are far less numerous nowadays than the “predatory ones”—or the personal ambitions of the founders. Our editorial experience with the journals *Brain and Language* and *Neuropsychologia*, as well as reviewing experience at many dozens of the top journals in neuroscience, psychology, and linguistics, non-specialized science journals, and applied journals in neurology, psychiatry, and speech and language, has given us insight into community needs, best practices, and common failings. Further, a younger generation of scholars, led by Dr. Jeremy Skipper of University College London and Dr. Uri Hasson of the University of Trento, provided us with creative insights into the future of publication and some of the advanced features that such modern publication might entail.

### Open Access

One of our primary goals with *Neurobiology of Language* is to enable all published manuscripts to be immediately available to anyone in the world who wants to read them, independent of subscription. The goal of ensuring such free and unfettered availability of all scientific work has led to an international effort to shift research-based publishing toward open access, which we were keen to support. Particularly in the United Kingdom and Europe, but also elsewhere in the world, many public and private funding agencies are requiring researchers to make their work freely available via this mechanism. Some of these agencies provide funding to cover the open access fees for publication of the research they fund, but in many cases this is (or soon will be) restricted to publications in fully open access journals, such as this one, and does not cover publication in hybrid journals.

### Plan S

One significant proponent of open access is the cOAlition S, an informal grouping of research funding organizations that have endorsed “Plan S,” an initiative for open access publishing that was launched in September 2018. Plan S requires that, from 2021, “all scholarly publications on the results from research funded by public or private grants provided by national, regional and international research councils and funding bodies, must be published in Open Access Journals, on Open Access Platforms, or made immediately available through Open Access Repositories without embargo” (https://www.coalition-s.org/). We have founded *Neurobiology of Language* within the open access framework and aim to be compliant with Plan S in this respect.

### Rigor, Reproducibility, and Replicability

*Neurobiology of Language* will work to ensure reproducibility and replicability, where “reproducibility means that someone other than a published study’s authors is able to obtain the same results using the authors’ own data, whereas replicability means that someone other than a published study’s authors is able to obtain substantially similar results by applying the same steps in a different context and with different data” (Aguinis, Cascio, & Ramani, 2017). To achieve this, all papers will be required to adhere to “best practices” in reporting experimental methods, as described for pre-clinical (animal) research (Steward & Balice-Gordon, 2014) and more recently, for imaging research, as commissioned by the Organization for Human Brain Mapping (OHBM; Nichols et al., 2017), and to submit all raw data to a central data repository for full open data access, such as those suggested by the Nature group (https://www.nature.com/sdata/policies/repositories) or those specific for human neuroscience data (e.g., several suggested databases are listed in the OHBM report (Nichols et al., 2017).

We are also initiating a collaboration - with the help of MIT Press and the other recently started open science journals at MIT Press (*Network Neuroscience*, *Open Mind*) - with the developers of BrainLife (https://brainlife.io/) to facilitate open data and replication via stored data, code, and an infrastructure that enables data analysis.

### Publishers

In addition to wanting a fully open access journal, we wanted a publishing house with focus on academic publishing, and it was important to us that the publishers were of the highest integrity and not-for-profit. This is particularly relevant in an era where thousands of illegitimate predatory journals (without peer review) have been founded on an open access model solely for profiteering. For these reasons, we chose the MIT Press as the publisher of *Neurobiology of Language*.

Established in 1962, the MIT Press is one of the largest and most distinguished university presses in the world and a leading publisher of books and journals at the intersection of science, technology, art, social science, and design. MIT Press books and journals are known for their intellectual daring, scholarly standards, interdisciplinary focus, and distinctive design. The MIT Press is known for publishing significant works by leading educators and researchers around the globe and achieving the broadest possible access, impact, and audience. MIT Press has been publishing leading edge journals for over 50 years, and launching new journals that have nurtured burgeoning areas of scholarship. In both form and content, the Press aims to push at the edges of scholarship, and has embraced the open access model for many of their journals. MIT Press was one of the first journal publishers in the world that moved subscription-based publications to completely open access publications.

### Costs

Although some funding agencies and academic institutions provide financial support to make publications open access, there is an increasing sense among academics of paying twice through the open access charge as well as the journal subscriptions being paid by their institutions to publishers for so-called hybrid journals. Discussions about open access fees and arrangements between publishing houses, governments, and universities are still ongoing and it is clear that publishing models are going to change.

The MIT Press has supported us in our aim to make open access publishing affordable and has worked with us to create a business plan based on article processing charges (APCs) well below the average. In addition, we received support from the National Science Foundation and the MIT Libraries to launch the journal. The editors-in-chief, the senior editors, and the reviewers are not paid for their service. We realized, however, that the journal needed the support of managing editor and some of the open access costs support that position.

With the society’s agreement, and with the strong support of its publication committee, headed by Matt Davis (Cambridge, UK), all profits for the next five years will be held by the journal to keep open access fees as low as possible. The costs to publish open access offer considerable value for money in particular for members of the society. Manuscripts where the first and last authors are current members have a discounted fee. In this way, we hope also to support the society by incentivizing membership by providing an additional benefit to members outside of attending the annual meeting and receiving the newsletter.

### Marketing

Starting a new journal involves more than good intentions, a great publisher, and dedicated editors and reviewers. More than anything it requires manuscripts, and this means that the community of scholars needs to know about us and to submit manuscripts for consideration. We are grateful to SNL for helping increase awareness, and to our editors for spreading the word. We also have an active Twitter account (@jneurolang) that currently has more than 1200 followers. In the current era, marketing critically involves social media and other online platforms, and for this, *Neurobiology of Language* needed an identifiable logo. With the help of MIT Press and the design firm Hecht/Horton Partners, we created an abstract logo that simultaneously suggested the concepts of left and right hemispheres, interhemispheric and longitudinal connections (using the diffusion tensor imaging color scheme), electroencephalographic and speech waveforms, and the double helix of DNA. This logo will increasingly be identified with the journal through its presence on social media, submission and publication platforms, and websites of MIT Press and SNL.

## FEATURES

### Double Blind Reviews

Authors are asked to submit articles without revealing their identities so that reviews can be conducted in a blinded fashion. Although we realize that absolute blinding is impossible, and in fact, some of the reviewed articles will have been already published on preprint sites such as bioRxiv or PsyArXiv, it is nevertheless possible to make the reviewing process less overtly personalized, since many reviewers will not know the identities of the authors, even if some would. By aspiring to blind reviewing, we aim to reduce the many types of implicit biases that often adversely affect the peer review process.

Reviewers also remain anonymous throughout the reviewing process unless they choose to reveal their identities by signing their review. At this point, we have chosen not to implement open reviewing, in which the identities of reviewers are revealed to the authors. There are arguments for and against this model, and we may review this and other reviewing practices as the journal matures.

### Registered Reports

The scientific enterprise as currently configured provides strong incentives to find positive results. It is often difficult to publish negative findings, even if such publication would save other investigators with similar well-motivated hypotheses significant time and effort. This problem was of such importance in the clinical world that the U.S. government mandated the registration of all large clinical trials and their universal access (clinicaltrials.gov). Further, many journals required that even small trials be registered for their results to undergo peer review.

An important mechanism for changing the incentive is to permit submission of papers consisting of only an introduction and extensive detailed methods, including planned data analyses. Such registered reports undergo rigorous review by a team of domain experts and statistics. Once a paper is accepted, the experiment can be executed, with a promise of publication of the results, however they turn out. This approach improves considerably the rigor and reproducibility of findings: Without the incentive for positive results, there is no incentive to try to extract weak “positive” findings from null results through the creative application of statistical methods. To quote the Center for Open Science (https://cos.io/rr/), registered reports “reduce the chances of low statistical power, selective reporting of results, and publication bias, while allowing complete flexibility to report serendipitous findings.” The *Neurobiology of Language* is keen to support registered reports.

### Consensus Reviews

During this first year of operation, the *Neurobiology of Language* has experimented with a novel form of reviewing, known as consensus reviewing, used very successfully by two open access journals that we have turned to for advice, *eLife* and *eNeuro*. About half of our senior editors are trialing this approach, and thus far, about half of all manuscripts have been reviewed under this framework. The goal of consensus reviewing is to simplify the process for authors by giving them a single aggregated and synthesized set of recommendations for manuscript improvement, leading to publication. Under this model, a senior (handling) editor assembles a reviewing team, composed of both senior scholars and early career researchers, and each team member performs an independent review (as under the more traditional model). What changes is in the subsequent step, when all members of the team participate in an online (private) discussion of their reviews, aiming to come to consensus on what the authors need to do to make their manuscript suitable for publication. One member of the team is then assigned the job of writing the consensus document that will be returned to the authors. Ideally, this document is not simply a rehashing of all the reviews but a succinct synthesis of them.

### Other Modern Publishing Features

In addition to open access, another driver of our goals, motivated by our colleagues, Drs. Jeremy Skipper and Uri Hasson, has been that the new journal would be modern and embracing of new innovations in publishing in terms of making data available flexibly and interactively.

A number of other modern publishing features are under consideration and will be trialed on an incremental basis over the coming years, based on community feedback and publisher operational imperatives. Our survey of the community did not find consensus on many of the possible features under consideration. These include (1) the identification of reviewers (attributed peer review) in published papers; (2) posting of reviews and responses online with manuscript publication; (3) post-publication commentary linked to manuscript publication; (4) pre-publication preprints for obtaining comments before/during peer review; (5) poster archives from conferences (especially SNL); (6) results-free reviewing (same as pre-registered reports but reviewed *after* the experiment has been run); (7) exploratory reports; (8) alternative metrics (view, view time, PDF downloads, Facebook, Twitter, etc. tracking); and (9) access credits or monetary rewards for reviews and comments.

### Availability Online

Authors submit their manuscripts for review in any format excluding any identifying information (see Double Blind Reviews section). Once a manuscript is provisionally accepted, authors are asked to submit a formatted version in which they are identified. The accepted manuscript is then sent for processing, and this version is made available electronically under “just accepted.” Thus, a digital object identifier (doi) is available and can be cited as soon as possible after acceptance. The version of record is updated as the article moves through the publication process; it is replaced by the uncorrected proof once it has been typeset, followed by the corrected proof. The final version (after the publication of this first issue) includes issue and page numbers for citation. The article is published under the Creative Commons CC BY 4.0 license (https://creativecommons.org/licenses/by/4.0/). Copyright of the published article is assigned to the Society for the Neurobiology of Language and Massachusetts Institute of Technology. Authors are allowed to self-archive the article on their website or in institutional repositories crediting the MIT Press and journal accordingly.

## EDITORIAL BOARD

### Senior Editors

We have had an overwhelming outpouring of support for editorial participation in the *Neurobiology of Language* from researchers at all levels of experience and accomplishment. The initial organization of the editorial board reflects primarily those individuals of the most experience or accomplishment or both, who serve as handling editors, coordinating the reviewing process in their specialized areas of knowledge.

### Editorial Consultants

All individuals who expressed an interest in the journal are increasingly participating in reviewing teams organized by the senior editors, and are valued members of these teams. Interested scientists who have not yet been invited to participate should remind the editors-in-chief and senior editors of their interest. We expect that the most active and responsive editorial consultants will in time, and commensurate with their own accomplishments, become senior editors in the coming years.

### Gender Balance

We wanted an editorial board that was balanced for gender, had representation from all corners of the globe, and had a breadth of expertise. We achieved the first goal and have equal numbers of men and women as senior editors. These people will serve for different terms in the first instance so it will be important that we maintain our current balance.

### Geographical Balance

In terms of our global reach, many continents are represented, but not all, and not sufficiently. The vast majority of our editorial board comes from Europe and North America, and we have some representation from Asia, Oceania, South America, and the Middle East. We have no representaiton from Africa. In the coming years, we will work to increase representation from global regions outside of Europe and North America.

## THE FIRST ISSUE

We wrote this editorial almost a year after we started accepting manuscripts. The first issue is a milestone for us and we would not have got here without the considerable efforts of our Managing Editor Dr. Salomi Asaridou. Salomi supports the co-editors-in-chief, senior editors, reviewers, and authors admirably and patiently as we all learn to navigate and test out new systems relating to the whole editorial process. We are extremely grateful to her and to all at MIT Press who have assisted us in these first 12 months.

This first issue of the first volume includes both the first manuscript submitted (Wilkinson et al., 2020) and the first manuscript accepted (Mercure et al., 2020). Coincidentally, these two studies focus on language development in infants, using EEG and functional near-infra-red spectroscopy (fNIRS), respectively. Fittingly perhaps, there were inevitable “teething troubles” with both manuscripts as we worked out the bugs in our editorial management processes. We are grateful to both sets of authors and reviewers for their early commitment to us and their patience with us.

The six publications in this first issue demonstrate the breadth of our field in terms of the methodology, including three papers using EEG, two using functional MRI, one each for fNIRS and regional gene expression profiling. They also demonstrate the depth and breadth of scientific inquiry: prediction and diagnosis of atypical language processing in autism spectrum disorders (Wilkinson et al., 2020), perception of spoken and signed languages in unimodal and bimodal infants (Mercure et al., 2020), distributional vector representations of word meaning (Sassenhagen & Fiebach, 2020), the effects of linguistic context and top-down prediction on comprehension (Brothers, Wlotko, Warnke, & Kuperberg, 2020), composition and word-order in sentence processing (Mollica et al., 2020), and the gene expression profiles of brain areas activated during language processing (Kong et al., 2020).

Several of the published manuscripts in this first issue underwent consensus reviewing. Feedback from authors was positive. For editors, the experience was more mixed. As expected, it was easier to generate a consensus document when there was a consensus view among reviewers and when reviews were written with this in mind. It can take a considerable amount of an editor’s or reviewer’s time but does make things clear for the authors. Other journals implementing consensus reviewing are compensating editors for their time. To keep costs low at the journal, we are not able to financially compensate editors or reviewers. Going forward, we need to weigh up the costs and benefits of consensus reviewing, along with other innovative features that we trial.

Anonymous author feedback: “I really liked the consensus review approach. It removes all the contradiction of one reviewer asking for more results while the other thinks your result section is way too long etc… It also makes reviewers a lot more anonymous than usual! And it probably removes some arguments that do not make sense. It was all in all a nice review experience. It improved the paper a lot and it was all fair comments.”

## CONCLUSIONS

*Neurobiology of Language* is a not-for-profit open access journal devoted to the neurobiology of language that incorporates modern publishing features, embodies the highest ethical standards, and facilitates data sharing, reproducibility, and replicability. With the support of SNL and our community of scholars, our aim is to keep pace with and adapt to the needs of our field and our broad readership of academics, professionals, clinicians, patients, and members of the public, all of whom contribute to our science in some way. With this editorial, we invite you to keep in touch, and tell us how we can improve this publishing venue further. We also invite you to continue your support of the journal by submitting your best work, editing and reviewing manuscripts in a timely manner, reading, sharing, and citing our publications, and generally promoting the *Neurobiology of Language* as an exciting new outlet through which we can disseminate our science. You can also follow us on Twitter @jneurolang.

Thank you for your support.
